# Human rights and the olympics: from an ideological paradox to a new anti-corruption legacy

**DOI:** 10.3389/fspor.2024.1365150

**Published:** 2024-09-18

**Authors:** Dikaia Chatziefstathiou, J. Simon Rofe

**Affiliations:** ^1^ Human Rights & Safeguarding Research Group, School of Psychology and Life Sciences, Canterbury Christ Church University, Canterbury, United Kingdom; ^2^ International Politics—Sports Diplomacy, University of Leeds, Leeds, United Kingdom

**Keywords:** major sporting events (MSEs), olympics, human rights, legacy, anti-corruption reform, sports diplomacy

## Abstract

This article aims to explore the evolution of the debates surrounding human rights in the discourse(s) of major sporting events (MSEs), particularly the Olympics. The paper will first analyse the western-centric origins of the “universality” of the Olympic philosophy and how it affected the protection and promotion of human rights, as well as addressing practical challenges or limitations faced. It will then focus on unpacking the elements of sportswashing and soft power in the multi-discursive context of sport where power, privilege, politics, and position are (re)produced. In doing so it draws upon the emergent field of Sports Diplomacy as the most appropriate explanatory framework to share in this discourse. Finally, it will explore the potential impact of anti-corruption reforms on the establishment of a meaningful human rights legacy.

## Introduction: human rights and major sport events (MSEs)

While major sporting events (MSEs) have the power to bring people together and promote teamwork, unity, and healthy competition, they also have a darker side when it comes to facilitating human rights abuses (1). A swather of research highlights the historical association of sporting activities with political protests that champion human rights, progressive socialism, and social inequality (2, 3). This suggests that MSEs can serve as a platform for political and/or human rights movements, to advocate for their ideals (4). There have been instances where MSEs and their governing bodies have come under scrutiny for their ties to corruption, cronyism, and the infringement of civil or human rights (5). This critical spotlight not only shines on the host nations of these events, but also on the governing bodies responsible for awarding hosting rights. Overall, MSEs have the opportunity to both promote and undermine human rights—potentially simultaneously—depending on how they are organised and conducted. In an era of increasingly open societies, it is important for individuals and organisations to closely monitor and hold accountable the host nations and governing bodies of MSEs to ensure ethical considerations and human rights are not neglected or violated. Sport provides a high-profile platform in the consciousness of a number of local, national and global publics for political protest and the raising awareness of human rights (6).

Research evidence suggests that staging MSEs has a range of impacts upon human rights (7). The focus has often been on forced evictions and displacement of populations (8), violation of labour rights (9), restrictions on freedom of opinion, expression and movement (10), direct political repression (11), and human trafficking (including sex trafficking) (12). Human Rights Watch Global Initiatives (13) identify five signature types of serious human rights violations: (1) forced evictions without due process or compensation; (2) abuse and exploitation of migrant workers; (3) the silencing of civil society and rights activists; (4) threats, intimidation, and arrests of journalists; and (5) discriminatory laws or actions by both host and participating countries (14). The focus is heightened in prominence in the period before MSEs with the Summer Olympic Games the most prominent of them.

This article aims to explore the evolution of the debates surrounding human rights in the discourse(s) of MSEs, particularly the Olympics. The paper will first analyse the western-centric origins of the “universality” of Olympism, the Olympic philosophy underpinning the Games, and how it affected the protection and promotion of human rights, as well as addressing any practical challenges and limitations. It will then focus on providing insight into the key concepts of sportswashing and soft power in the multi-discursive context of sport where power, privilege, politics, and position are (re)produced. Finally, it will explore the potential impact of anti-corruption reforms in mega-sporting events on the establishment of a meaningful human rights legacy. In addressing these, the article draws on the analytical frameworks of Sports Diplomacy, as opined by Stuart Murray and J Simon Rofe, and utilised to explore the following three key themes:

(a) The Olympic philosophy and its ideological paradoxes in relation to human rights values;
(b) The phenomenon of “sportswashing”, and consideration of Soft Power with their implications for human rights in the Olympics and other MSEs;
(c) The potential for anti-corruption reforms in MSEs to leave a lasting human rights legacy.

### A. Olympism and its ideological paradoxes

Debates surrounding human rights and the Olympics have their antecedents in the ideological paradoxes of Olympism. Leading figures of the Olympic movement, such as the founder of the Olympics Baron Pierre de Coubertin, talked about universalism and internationalism; “the power that Olympism retains in the face of the lizards proclaiming its imminent or more gradual collapse derives from its most deeply human, and therefore universal, aspects” [(15): 517]. However, the ideological constituents of Olympism also served to undermine human rights, particularly in the early years of the Olympic movement. The exclusion of women and the provision of a podium for the celebration of competitive, physical masculinities raising questions about the “universal” ideals of Olympism. Messages underpinned by western-centric imperialist forces with racial categorisations and discriminations, sharing characteristics with Social Darwinism, were integral to the operationalised Olympism of the early century. Ethnic and racial discrimination were evident despite espoused ideals of equality and fraternity, especially in relation to competitors from Africa and Asia. As such the modern Olympic Games have been conceived and dominated by western society and have largely reflected elite and Eurocentric values (16). As Chatziefstathiou and Henry (17) have argued, a Eurocentric logic was apparent and dominant in the discourses of the modern Olympic movement. De Coubertin's words from 1923 provide an insight:

“Let us think however, for a moment, of what is troubling the African soul. Untapped forces-individual laziness and a sort of collective need for action-a thousand resentments, and a thousand jealousies of the white man” [(18): 497].

While of their time, for many authors and peoples around the world then and subsequently, Coubertin's philosophy did not promote “universal” human rights values, rather it applied to a time specific Eurocentric context where representatives from “oppressed nations” were absent from the Games (16). It should be noted that interpretative understanding is the end-product of a hermeneutic process in which “the researcher relates the literal meanings to the contexts in which they were produced in order to assess the meaning of the text as a whole” (19); cited in [(20): p. 30]. Taking into consideration the social context surrounding a document is essential to grasping the significance of the document alone (21). Similarly, Hodder (22) argues that the context of the text is crucial in understanding its meaning, as a text can say many different things in different contexts. Therefore, past and present meanings of the same text should be questioned, as values and ideas are contested over time and anachronistic statements may distort the meaning of the text in its specific historical, political, and socio-cultural context. Altheide (23) argues that the “emergence of meaning” should be sought through a constant comparison and investigation of documents over a period of time following a reflexive movement between the content of the document, the theoretical assumptions underpinning the study, and the broader historical context.

As Lekarska (24) argued, Coubertin's ideas should be seen as part of a specific historical context, that of imperialism. And she pointed out that even though amateurism and women's exclusion are outdated values, the values of internationalism, excellence and moral development through sport remain important constituents of Olympism. In similar vein, Neverkovich (25) noted that the Olympic idea and the form of its realisation have become part of modern times, enriched and modified by social and historical experience (Neverkovich). Regarding the issue of amateurism, Parry (26) argued at the 28th IOA Session,

“If it is indeed true that the commitment to amateurism is dying, it is just as well that amateurism turns out to have been a historically specific element, which is simply becoming an outmoded factor. If, on the other hand, it had been the central universal value of Olympism, this would be indicating that Olympism itself is dying, since its central value is. Olympism is alive and well, but amateurism is not. This shows that there must be some other source of the values of Olympism than amateurism”. [(26): lines 458–472, emphasis added].

Nevertheless, although Coubertin's Olympism sooner or later gradually adapted to the emergent socio-political, and cultural values, the Olympic Movement has always been criticised for Western-centrism, in particular Eurocentrism. Following the end of the Cold War, it has been suggested that the domination of the Olympic Movement by the West increased as signified by political power, economic interests, and the origins of sport on the programme. Donnelly (27) argued that the Olympics became a form of global sport monoculture. In a press-conference in Barcelona the IOC spokeswoman Michele Verdier answered a question regarding the absence of women athletes in the Olympic delegations of many Islamic countries creating “a storm” with her answer [(28): p. 71]. In MacAloon's viewpoint, Verdier suggested that the IOC was not prepared to more strongly intervene because gender issues were “a matter of religion and custom” in these societies. The stated concern was less with the danger of feminist imperialism than with religious imperialism, reflecting the special and especially ambiguous status of “religion” as a category in European logic (p. 71). MacAloon (28) argued that Olympism is ideologically and practically engaged in the production of transpersonal, transnational, and pan-human identities (29). Olympic internationalism is thus based on the Eurocentric conception that there exists a world of universal truth irrespective of human differences in culture and tradition (30). The Olympic values are, thus, largely considered as western moral ideals, principles of western liberalism (31). Guttmann [(32): p. 72] argues that “the root difficulty” of the Games “is that modern sports, like the universalistic political ideals institutionalised in the Olympic Games, are themselves a product of western civilisation”. The value of true universalism has thus bee challenged from the domination of western and Eurocentric logic in the orientation and later practices of the Olympic Movement.

### B. Sports diplomacy: sportswashing and soft power in the history of the Olympics

In an era of Eurocentric colonialism and paternalism, sport served as “an economy of affect through which power, privilege, politics, and position are (re)produced” (33). Sport's use as a vehicle for power and politics has long been explored. The framework of Sports Diplomacy [(34, 35) & 2018] that has become a key aspect of discourse in the realm of sport offers a meaningful way to consider sportswashing and soft power alongside other dimensions. Murray's definition considers Sports Diplomacy as a relatively “new term that describes an old practice: the power of sport to bring people, nations, and communities closer together via a shared love of physical pursuits”; while Rofe's analysis points to Sport Diplomacy as an “explanatory overlay to the network of evolving networks within the worlds of sport and diplomacy.” (34, 36). Arising from these two accounts, and an increasingly large body of additional sources representing diverse understandings, there is scope to understand to a greater degree both sportswashing and soft power. In recent times, and often with negative connotations, the relationship between power and politics is being referred to as “sportswashing”. Building upon longer held practices, it encompasses the use of the Olympics and other MSEs to enhance a host nation's perceived prestige and promote economic/political/cultural progress. Boykoff (37) defines sportswashing as “a phenomenon whereby political leaders use sports to appear important or legitimate on the world stage while stoking nationalism and deflecting attention from chronic social problems and human-rights woes on the home front” (p. 342). This practice can be observed in both authoritarian and democratic political contexts and involves targeting both domestic and international audiences—particularly drawing upon the concept of Public Diplomacy (38). Nye (39) argues that soft power is the ability to co-opt rather than coerce. It is about shaping the preferences of others through appeal and attraction for instigating change. The concept of soft power provides scope for sportswashing. As such sportswashing operates as a form of social relationship, entangling various audiences and influencing public perception through the integration of sports and culture for specific political ends. Through this process, sportswashing not only reflects certain historical narratives but also plays a role in shaping future perceptions and national identities (37).

Though the term “sportswashing” was coined in the 21st Century, it is not a new phenomenon. We can detect examples of sportswashing in the ancient Olympics in Greece. In 416 BCE, during the war between Athens and Sparta, Alcibiades, a young Athenian politician on the rise, entered many chariot teams to the Olympics. They achieved several victories that were used to distract the public from the war defeats (40, 41). Alcibiades referred to those sports successes in his speeches as evidence of power and to persuade Athenians to invade Sicily (42). Elements of sportswashing were also present in the 1936 Berlin Summer Olympics as the event highlighted the ethos of the ruling National Socialist Party under Chancellor Adolf Hitler. Although Hitler was originally opposed to the idea of Germany hosting the Games, he came to understand that staging such an international event would provide an excellent opportunity to project Nazi propaganda (43). Recognising how the sporting event can shaped, the Nazi regime contributed a novelty to the Berlin 1936 Olympiad: the torch relay from Olympia in Greece to Berlin. It was a novelty introduced by Carl Diem who believed that “the aim of the relay was to emphasise the spiritual vitality and moral value of the Games both in ancient and modern times, and to show that the same idealism fills the youth of today” [(44): 46]. His focus was on the youth of the world:

“The fire having once been carried from Olympia to Berlin for the 1936 Games, the idea of such link refused to die away. For it symbolises devotion to the common ideal, which the Olympic celebration embodies, and the wish to implicate not only the actual competitors in the Games but the still uncommitted youth of the world”. [(45): p.77]

In relation to the torch relay, Roche (46) argues, “This was a product of Diem's long-standing and probably sincere sporting and Hellenic idealism. However, in this new Nazi context of the mid-1930s it inevitably carried with it some shadowy and suspect connotations and implications”. Olympia had been used as a site for the ancient Greek Games for around a thousand years, representing, as such, a kind of “a thousand-year civilisation”. This helped to inspire Hitler and his “vision” of a “Thousand Year Reich” [(46): p.117]. *The New York Times* described Hitler as “the new Caesar of this era” who “was receiving the plaudits of a league far removed from politics, a league of peaceful sport to which he had become the proud host” [(47): p.1].

The IOC's association with the Nazi regime drew contemporaneous criticism, for resisting calls for boycotts on the basis of the regime's sport policies and thus the Games themselves, and for allowing the event to be draped—literally in terms of dressing the stadium—Nazi symbolism and propaganda., Since 1936 criticism has continued. The British sport journalist and historian John Rodda argues that the IOC was “completely insulated from political events within Germany and the strong overtones produced at the Berlin Games” [(48); quoted by (46): p.119]. Hoberman (49) regards this period of the Olympic Movement as one of, at best fascist collaboration or, at worst one of ideological domination by fascism. Additionally, Lucas (50) also questioned the nature of the Olympic Movement in this period, not being able to understand many of the “bizarre” decisions that were taken (p. 135). These views speak to the belief present amongst many in the Olympic movement during the 20th Century that politics and sport could be separated. This view, non-sensical to the long history of human activity, became almost messianic as it was espoused by IOC President 1952–1972 Avery Brundage when he suggested in 1959: “The moment politics are permitted in Olympic affairs, the Games are finished.” Brundage extoled what Lincoln Allison calls the “Myth of autonomy”: that sport is autonomous from the society in which it sits [Brundage, in Sports Illustrated 15 June, 1959; (51)].

Yet, to quote Brundage again in the aftermath of the 1972 Munich Terror attacks “The Games *must* go on” and so does the relationship of politics and sport in sportswashing terms. The 2014 Winter Olympics in Sochi, Russia have been scrutinised for giving the opportunity to Russia President Vladimir Putin to obscure changes in law that disadvantaged lesbian, gay, bisexual, transgender individuals (52). More broadly, MSEs are often employed by governments to support their narratives in relation to social phenomena such as gentrification, homelessness and citizens’ surveillance. Examples in the twenty-first century include the FIFA Men's World Cup, in South Africa in 2010 and four years later in Brazil, and the 2016 Summer Olympic and Paralympic Games. Indeed, it is often part of the rationale for the award of the Games. For instance, Los Angeles Mayor Eric Garcetti used the scourge of homelessness to sportswash, stating that “I'm confident that by the time the Olympics come, we can end homelessness on the streets of LA” [(37): p.343].

### C. Anti-corruption reforms: a new human rights legacy

Twenty-first Century decisions by the IOC have been seen as “bizarre” or inappropriate by many advocates of the established human rights regime. For example, the democratic and peace-making profile of the IOC has been undermined by the selection of Beijing as a host city for the 2008 Summer Olympics, the 2014 Winter Olympics in Sochi, and the return to Beijing for the 2022 Winter Olympics. Despite the 1989 Tiananmen Square massacre and allegations of human rights abuses by the Chinese state, the IOC awarded the 2008 Games to Beijing (46). Seven years later the IOC also awarded the 2022 Winter Olympics to Beijing despite failed promises from the 2008 Beijing Olympic Bid Committee to enhance social conditions, including education, health and human rights (37, 53). In the lead up to the 2016 Rio Summer Olympics, reports of the demolition of slum communities to creating spaces for Olympic venues were deemed consistently newsworthy. The violation of labourers’ rights during the works of the infrastructure for the 2022 Men's FIFA World Cup in Qatar was also widely reported. Lagon and Nasielski (54) argued that the IOC's and FIFA's approach in the early twenty-first century has made the games increasingly synonymous with financial mismanagement, autocracy, and the systematic violation of human rights; entirely at odds with both the longstanding, and recently professed goals of the both organisations.

A counter-narrative has emerged which places an increasing focus on the human rights legacy and anti-corruption reforms prompted by MSEs, especially the Olympics. In the 24th Session of the United Nations Human Rights Council (55) a resolution on “promoting human rights through sport and the Olympic ideal” was adopted (55). In December of 2014, the International Olympic Committee (“IOC”) launched the “Olympic Agenda 2020”, a set of 40 recommendations to safeguard the Olympic values and strengthen the role of sport in society (56). In 2017, the IOC decided to move forward with the implementation of the Olympic Agenda 2020 and added new human rights contractual provisions to the Host City Contracts (“HCCs”) and its respective documentation, commencing from Paris 2024. The key provision inserted in new HCCs regarding human rights is Section III (Core Requirements), Article 13.2(b) which states as follows:

“[…] The Host City, the Host NOC and the OCOG shall, in their activities related to the organisation of the Games: b. protect and respect human rights and ensure any violation of human rights is remedied in a manner consistent with international agreements, law and regulations applicable in the Host Country and in a manner consistent with all internationally recognised human rights standards and principles, including the United Nations Guiding Principles on Business and Human Rights, applicable in the Host Country.” (57).

The above is also included in Section III, article 15.2(b) that addresses more specifically compliance with laws and regulations of the Host Country as well as international agreements regarding planning, construction, protection of the environment, health and safety, labour and working conditions and cultural heritage (57). The same clause was included in the Candidature Questionnaire, obliging the government of the Candidature Country to guarantee that key measures will be in place to ensure that every activity related to the organisation of the Games complies with the provision (58). Also, another clause was included to the IOC Supplier Code stating, “suppliers shall respect international proclaimed human rights and ensure that they are not complicit in human rights abuses” (59).

This shift in focus is a direct response to the growing scrutiny and criticism that the IOC faced, and the growing awareness of the negative impacts that hosting MSEs can have on human rights. It could be argued that the new provisions impose a positive obligation on the parties to protect human rights and remedy any violations. However, one key problem with these contractual provisions and guarantees is that new clause limits the Human Rights obligations to those applicable in the host country, and *not all* Host Countries are bound by the same Human Rights obligations under National or International Laws (60). This could hinder the offer of protections and remedies even available for individuals involved in the organisation of the games (61). Moreover, the Host City Contract states that the IOC will establish a reporting mechanism to address the Human Rights Obligations of the Host City and ensure compliance (57). This reporting mechanism is to be set up by the Coordination Commission. The Coordination Commission includes representatives of the IOC, the IFs, the NOCs the athletes and experts. As defined in the Olympic Charter, the Coordination Commission's mandate is to: monitor the progress of, and provide guidance to the Organising Committee of the Olympic Games (OCOG), with respect to the planning, organisation, staging and financing of the Olympic Games, including in relation to collaborating with the relevant public authorities; conduct on-site inspections of competition, training venues and other facilities; report to the IOC Executive Board on the status of preparation of the Olympic Games, particularly with regard to progress, challenges and risks; after the Olympic Games, to produce a report relating to the organisation of the Olympic Games for the IOC Executive Board and IOC Session. However, the Commission typically only visits the host cities once or twice a year to check on progress (62). As Prince (61) argues, this reporting mechanism provides no oversight beyond what the parties to the Host City Contract take up responsibility for or what is examined during a Coordination Commission visit, which could be very ineffective if the host city is aware of when they are coming. Most importantly, there is no remedy or enforcement mechanism for third parties under the contract, just that the Host City, the NOC and OCOG should remedy any violations (57). The provision does not indicate whether they should set up an oversight system to handle complaints and ensure remedies, or if they would fulfil these duties by providing information on where parties whose rights have been violated can file a lawsuit. Consequently, “this leaves the Host City, the NOC and OCOG a bit at loose ends” [(61): p.2].

Boykoff (63) recently claimed that our times constitute “an enormously important moment when it comes to the relationship between the Olympics Games and human rights” as he urged scholars to “peer behind the shiny scrim of the Olympics and seriously scrutinise the effects that staging the Games has on host cities”. Byrne and Lee Ludvigsen (64) also caution against an uncritical praise of institutional embrace of international human rights standards by the IOC. As they claim, a human rights reform in the Olympic arena will always face limitations due to the neoliberal underpinnings of the Olympics and other MSEs. They argue that whilst the IOC's embrace of human rights remains a positive development, “Olympic “legacies” do not always materialise that the celebrated reforms that have thus far taken place, are subject to critical and ongoing scrutiny—from academics, advocacy groups, journalists, and practitioners—to determine their human rights compatibility with internationally protected standards” (p. 14).

## Conclusions: future opportunities

MSEs have the potential to leave a significant positive legacy, including promoting physical activity, sports participation and a festival effect (65, 66). However, it is essential to acknowledge the potential negative associations and impacts, such as the ideological paradoxes of the Olympic philosophy and human rights abuses (17, 67, 68). The potential negative impacts of MSEs, such as human rights violations and the displacement of vulnerable communities, need to be considered as well as the effectiveness of current anti-corruption strategies in each sport (68, 69).

The exposure of corrupt practices and the demand for accountability from the public and global anti-corruption movements, often manifest in the form of sponsors, put pressure on sports federations and planning bodies to prioritise human rights and combat corruption. This has led to the implementation of stricter ethical codes and regulations, as well as increased transparency and oversight in the bidding and organizing processes of these events. Philippou (70) underscores the need for anti-corruption measures, which are crucial for ensuring that human rights are upheld in the organisation of these events. Host nations are now being evaluated not only on their ability to provide world-class venues and infrastructure but also on their track record of human rights and their commitment to anti-corruption measures; a worthwhile outcome in the authors’ opinion. Amis (71) and Heerdt (60) both highlight the need for multi-stakeholder collaboration and the inclusion of human rights provisions in bidding and hosting agreements. Heerdt (72) further emphasises the importance of a shared responsibility approach, which involves preventative and retrospective measures, as well as collaborative remedy.

As such ensuring good governance and preventing corruption in both the planning and delivery of MSEs demonstrate the potential for non-sports legacies in MSEs (73). Acknowledging these potentials is key to the framework Sports Diplomacy provides. The role of public finance in MSEs should be reconsidered to reduce the opportunities for corruption (73), while the influence of civil society and international organizations acknowledged in pushing for strict reforms and combating corruption is crucial (74). The formation and implementation of an effective anti-corruption system requires clear interaction of law enforcement institutions at the local, national and transnational levels (75). For instance, the United Nations Convention on the Rights of the Child (UNCRC), drafted in 1989, outlines child rights protection measures that should be implemented by all signatories. However, as tends to happen with many rights conventions signed by nation states, the general commitments made “are rarely specific enough to address all eventualities” [(7): 2]. So, “while the UNCRC states that host nations should consider the rights of the child when bidding, planning for, and delivering their MSEs, evidence suggests that insufficient consideration is given to child rights in policies guiding the Games” [(7): 2] [(76, 77)]. Human rights infringements such as child rights need to be embedded more specifically in MSEs policies for each hosting nation, and they should also be further addressed in academic debates (7). These challenges demonstrate that there is still a gap in empirical evidence and knowledge regarding anti-corruption issues associated with the organization of MSEs (70). Organisations such as the Centre for Sport and Human Rights provide an important focus to highlighting the relationship between different dimensions of the Human Rights landscape and sports opportunity to shape it. The launch in June 2024 of the Human Rights Playbook: aimed at empowering “sports bodies worldwide to make robust human rights commitments” speaks to the potential here moving forward (78).

By implementing effective anti-corruption reforms in MSEs, both the IOC and other International Sporting Federations (ISFs) such as FIFA; and host countries can create a foundation for a meaningful human rights legacy (79). Spalding (80) argues that a transformation is happening, even if it has been overlooked, in sharing positive human rights legacy through an improved culture of good governance. In prioritising transparency and accountability, host countries can demonstrate their commitment to upholding human rights principles. Importantly by addressing human rights concerns during the event planning and implementation stages, host countries can foster a positive and inclusive environment for all participants, contributors, contractors, and sponsors, regardless of their background or nationality. There is potential for anti-corruption reforms to leave a lasting human rights legacy in MSEs but requires a comprehensive and coordinated approach from all involved parties. Efforts to establish a new human rights legacy and anti-corruption reforms in MSEs should focus on addressing these issues.

The new legacy of human rights and anti-corruption reforms in MSEs are a testament to the evolving nature of sports as a force for social change. By embracing human rights the Olympic Games, and other MSEs provide an opportunity to restate their place in the public's conscience as catalysts for positive change. Furthermore, the new legacies’ calls for scrutiny, demonstrate the network of networks of Sports Diplomacy: i.e., the manner in which sports activities through their convening power—to “attract”—demonstrate impacts in other parts of global diplomatic practice across human rights, education, health and well-being, and sustainability.

As highlighted earlier, what is absent from the HCC is clear guidance on how the protection of these rights should be secured and how remedies can be made readily available and accessible for those whose rights have been violated, who are often equity deserving groups with limited resources and access to legal support. Future work in this area should explore the effectiveness of existing mechanisms in preventing human rights violations during MSEs that can advance our practical and academic understanding of human rights and sport.
